# MALT1 Controls Attenuated Rabies Virus by Inducing Early Inflammation and T Cell Activation in the Brain

**DOI:** 10.1128/JVI.02029-17

**Published:** 2018-03-28

**Authors:** E. Kip, J. Staal, L. Verstrepen, H. G. Tima, S. Terryn, M. Romano, K. Lemeire, V. Suin, A. Hamouda, M. Kalai, R. Beyaert, S. Van Gucht

**Affiliations:** a National Reference Center of Rabies, Viral Diseases, Communicable and Infectious Diseases, Scientific Institute of Public Health, Brussels, Belgium; b Center for Inflammation Research, Unit of Molecular Signal Transduction in Inflammation, VIB, Ghent, Belgium; c Department of Biomedical Molecular Biology, Ghent University, Ghent, Belgium; d Scientific Service Immunology, Communicable and Infectious Diseases, Scientific Institute of Public Health, Brussels, Belgium; e Laboratory of Virology, Department of Virology, Parasitology and Immunology, Faculty of Veterinary Medicine, Ghent University, Ghent, Belgium; University of Kentucky College of Medicine

**Keywords:** ERA, MALT1, immunity, neuroinflammation, rabies virus

## Abstract

MALT1 is involved in the activation of immune responses, as well as in the proliferation and survival of certain cancer cells. MALT1 acts as a scaffold protein for NF-κB signaling and a cysteine protease that cleaves substrates, further promoting the expression of immunoregulatory genes. Deregulated MALT1 activity has been associated with autoimmunity and cancer, implicating MALT1 as a new therapeutic target. Although MALT1 deficiency has been shown to protect against experimental autoimmune encephalomyelitis, nothing is known about the impact of MALT1 on virus infection in the central nervous system. Here, we studied infection with an attenuated rabies virus, Evelyn-Rotnycki-Abelseth (ERA) virus, and observed increased susceptibility with ERA virus in MALT1^−/−^ mice. Indeed, after intranasal infection with ERA virus, wild-type mice developed mild transient clinical signs with recovery at 35 days postinoculation (dpi). Interestingly, MALT1^−/−^ mice developed severe disease requiring euthanasia at around 17 dpi. A decreased induction of inflammatory gene expression and cell infiltration and activation was observed in MALT1^−/−^ mice at 10 dpi compared to MALT1^+/+^ infected mice. At 17 dpi, however, the level of inflammatory cell activation was comparable to that observed in MALT1^+/+^ mice. Moreover, MALT1^−/−^ mice failed to produce virus-neutralizing antibodies. Similar results were obtained with specific inactivation of MALT1 in T cells. Finally, treatment of wild-type mice with mepazine, a MALT1 protease inhibitor, also led to mortality upon ERA virus infection. These data emphasize the importance of early inflammation and activation of T cells through MALT1 for controlling the virulence of an attenuated rabies virus in the brain.

**IMPORTANCE** Rabies virus is a neurotropic virus which can infect any mammal. Annually, 59,000 people die from rabies. Effective therapy is lacking and hampered by gaps in the understanding of virus pathogenicity. MALT1 is an intracellular protein involved in innate and adaptive immunity and is an interesting therapeutic target because MALT1-deregulated activity has been associated with autoimmunity and cancers. The role of MALT1 in viral infection is, however, largely unknown. Here, we study the impact of MALT1 on virus infection in the brain, using the attenuated ERA rabies virus in different models of MALT1-deficient mice. We reveal the importance of MALT1-mediated inflammation and T cell activation to control ERA virus, providing new insights in the biology of MALT1 and rabies virus infection.

## INTRODUCTION

The paracaspase MALT1 (mucosa-associated lymphoid tissue lymphoma translocation gene 1) is an intracellular protein that mediates nuclear factor κB (NF-κB) and p38/JNK mitogen-activated protein kinase signaling in response to multiple stimuli, including antigen receptor activation in lymphocytes, dectin-driven dendritic cell activation, and thrombin- and angiotensin-induced activation of fibroblasts and endothelial cells (1). In resting cells, MALT1 is constitutively associated with the caspase recruitment domain (CARD)-containing protein BCL10 and, upon stimulation, forms a complex with one of the CARD-containing proteins, CARD9, CARD10 (CARMA3), CARD11 (CARMA1), or CARD14 (CARMA2), depending on the cell type and stimulus (2). The CARD-BCL10-MALT1 (CBM) complex acts as a scaffold for other proteins, including TRAF6, which then mediates the activation of NF-κB and p38/JNK (3). In addition to its scaffold function, MALT1 holds proteolytic activity that cleaves a limited number of proteins at well-defined sites (4). MALT1 substrates include among others, NF-κB family members (5), ubiquitin regulatory enzymes (6–8), and RNase and mRNA-destabilizing proteins (9, 10). MALT1 proteolytic activity is not essential for NF-κB or p38/JNK activation but further fine-tunes gene expression that contributes to immune cell activation, proliferation and survival. Because of its major role in lymphocyte activation and proliferation, targeting MALT1 proteolytic activity via small compound inhibitors is currently of high interest for the treatment of several autoimmune diseases and lymphoma (11–13).

Genetic mouse models have provided key insights into the biology of MALT1. MALT1^−/−^ mice are viable, fertile, and born at the expected Mendelian ratios (14, 15). Their total number of T cells and the distribution of CD4^+^ and CD8^+^ T cells in the spleen, lymph nodes, and thymus is comparable to those of wild-type mice, but T cell activation, proliferation, and interleukin-2 (IL-2) production are decreased, leading to a lower number of activated T cells in the periphery (15). MALT1 is also required for the development of regulatory T cells in the thymus (16, 17), as well as the development of marginal-zone and B1 B cells (14, 15), but is dispensable for the development of normal B2 B cells. MALT1^−/−^ mice present impaired IgM- and CD40-induced proliferation and lower basal serum immunoglobulin levels, with IgM and IgG3 showing the most pronounced reduction. A limited number of patients carrying MALT1 mutations leading to severe immunodeficiency have been reported (18–20), further illustrating the key role of MALT1 in innate and adaptive immunity. Finally, knock-in mice expressing a so-called “protease-dead” mutant of MALT1 in which the catalytic activity is disrupted, but which can still function as a scaffold for NF-κB activation, mimic all immune defects that are observed in full MALT1^−/−^ mice, although to a lesser extent. Surprisingly, these mice spontaneously develop autoimmunity because of a deregulated effector T cell response (reviewed in reference 1).

Although genetic studies have indicated that MALT1 deficiency may lead to severe immune defects, promising results upon pharmacological targeting of MALT1 in preclinical mouse models of multiple sclerosis (12) and ABC-type diffuse large B cell lymphoma (11, 13), without obvious side effects, have stirred a lot of interest in the therapeutic targeting of MALT1. However, a better understanding of the role of MALT1 in different tissues and under different conditions is essential. Previous studies already shown that MALT1 deficiency protects against experimental autoimmune encephalitis (EAE) (12, 16, 17), illustrating a role for MALT1 in the central nervous system (CNS). In the present study, we focused on the impact of MALT1 deficiency on infection with a neurotropic virus. More specifically, we investigated whether MALT1 deficiency affects the virulence of an attenuated rabies virus.

Rabies virus is a highly neurotropic negative single-stranded RNA virus that belongs to the genus Lyssavirus, family Rhabdoviridae (21). Rabies virus normally causes a highly lethal infection of the brain, which escapes control from the immune system (22). The virus still kills at least 59,000 humans each year and remains an important public health problem (23). A small number of humans have survived clinical rabies virus infection (24, 25), suggesting that, at least under certain conditions, the immune system can clear rabies virus from the brain. Evelyn-Rotnycki-Abelseth (ERA) virus is a highly attenuated rabies virus laboratory strain that was first described in the 1960s (26, 27). In contrast to virulent rabies virus strains, ERA virus induces a benign nonlethal infection of the nervous system that is associated with an efficient immune response (28). The ERA strain has therefore been used as a live vaccine for oral immunization of wildlife (29). Since the ERA virus strain causes a nonlethal brain infection in mice and can mount protective immunity against rabies, it allows study of the mechanisms by which the host can control rabies virus in the brain.

The role of T cell immunity in the control of rabies virus infection has been thoroughly documented (30–32). To study the impact of MALT1 activity on rabies virus infection, we compared the clinical and immunological effect of ERA virus infection in MALT1^+/+^ and MALT1^−/−^ mice, as well as conditional MALT1^−/−^ mice, lacking MALT1 in specific cell types such as T cells, neuroectodermal cells, or myeloid cells. We also examined the effect of treatment of mice with mepazine, a phenothiazine derivative and reversible MALT1 protease activity inhibitor (33). Our results demonstrate that MALT1 plays an important role in the control of infection by attenuated rabies virus in the CNS of laboratory mice by inducing neuroinflammation and by recruiting and activating CD8^+^ and CD4^+^ T cells within the brain in the early phase of infection.

## RESULTS

### Attenuated ERA virus becomes virulent in MALT1^−/−^ mice.

**FIG 1:**
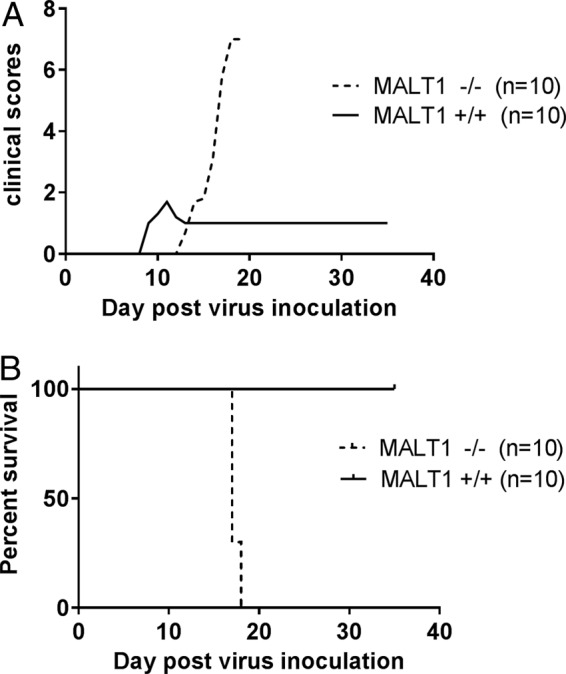
MALT1 is critical to control the pathogenicity of ERA virus. MALT1^−/−^ (*n* = 10) and MALT1^+/+^ littermates (*n* = 10) were infected intranasally with ERA virus. Clinical symptoms (A) and survival rates (B) were assessed. All MALT1^−/−^ mice developed severe disease and had to be euthanized. MALT1^+/+^ mice developed only mild symptoms. The results shown are representative of two independent experiments.

**FIG 2:**
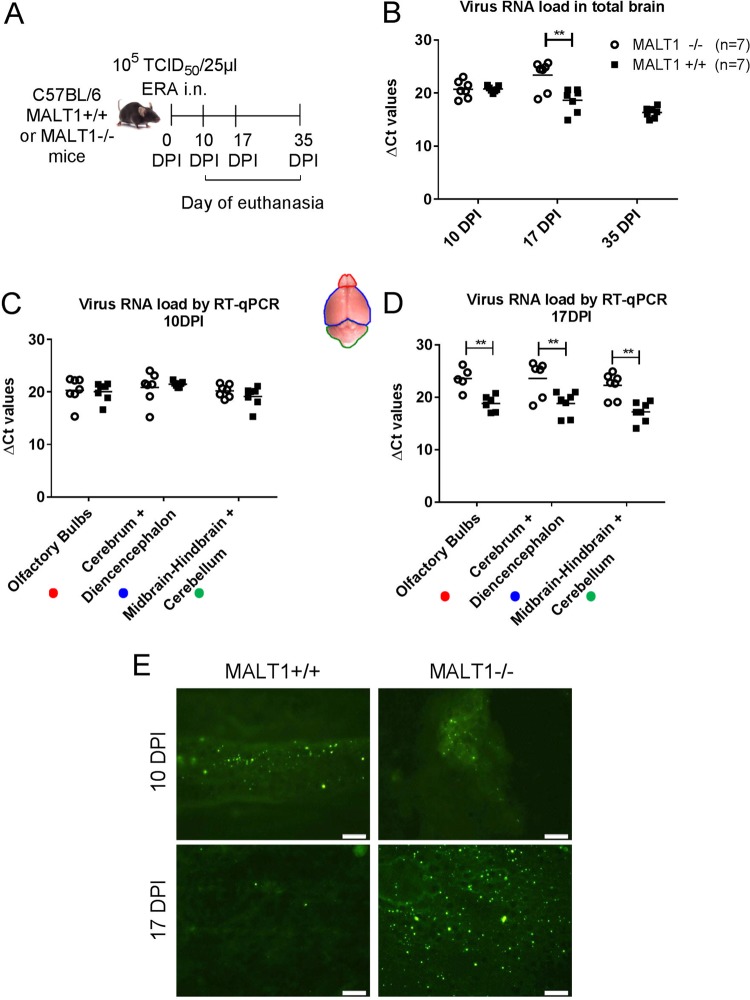
Virus spread in the brains of MALT1^−/−^ and MALT1^+/+^ mice after intranasal inoculation. (A) Schematic overview of the experiment. Mice were inoculated intranasally with ERA virus and sacrificed at 10, 17, and 35 dpi. (B) Profile of viral RNA load in total brain determined by RT-qPCR. (C and D) Profile of viral RNA load in different parts of the brain (**, *P* ≤ 0.01). (E) Immunofluorescence staining for viral nucleocapsid in the brain tissue. At 10 dpi, green fluorescent spots indicate the abundant spread of virus in the brain of MALT1^−/−^ and MALT1^+/+^ mice. At 17 dpi, only small amounts of viral antigens were still visible in MALT1^+/+^ mice, whereas viral antigens were still abundant in the brains of MALT1^−/−^ mice. These results are representative of three mice per time point and per genotype. Scale bars, 20 μm; magnification, ×40.

Intranasal inoculation of ERA virus in wild-type C57BL/6 mice resulted in a clinically benign infection with, as expected, complete survival (34) (Fig. 1). Minor clinical signs were observed at day 10 (rough hair coat and a slightly reduced reactivity), and the general appearance (rough hair coat) improved rapidly by 11 days postinoculation (dpi). Nevertheless, most mice remained slightly less reactive throughout the follow-up period (35 dpi), resulting in a clinical score of 1. In contrast, ERA virus-inoculated MALT1^−/−^ mice presented the first clinical signs at around 15 dpi and developed severe disease, characterized by limb paralysis and depression, requiring euthanasia at 17 or 18 dpi. These data indicate that MALT1 is necessary to control infection with ERA virus. Viral loads were analyzed in total brain by quantitative reverse transcriptase PCR (RT-qPCR) and fluorescent antigen test (FAT) and in the olfactory bulbs, cerebrum, and cerebellum by RT-qPCR (Fig. 2A to D). At 10 dpi, viral loads were similar in the brains of MALT1^−/−^ and MALT1^+/+^ mice (Fig. 2C). At 17 dpi, which corresponds with the end stage of disease in MALT1^−/−^ mice, a significantly higher viral load was observed in the different parts of MALT1^−/−^ mice compared to MALT1^+/+^ mouse brains (Fig. 2D). Fluorescence staining of the viral N protein yielded a similar signal in MALT1^−/−^ and MALT1^+/+^ brains at 10 dpi, whereas at 17 dpi more fluorescent foci were observed in MALT1^−/−^ mouse brains (Fig. 2E), in agreement with the results obtained by RT-qPCR. These results show that deficiency of MALT1 leads to a progressive and lethal increase of ERA viral load in the brain, resulting in severe clinical signs.

### Antiviral and inflammatory gene expression is severely disrupted in the brain of MALT1^−/−^ mice at the presymptomatic phase of infection.

**FIG 3:**
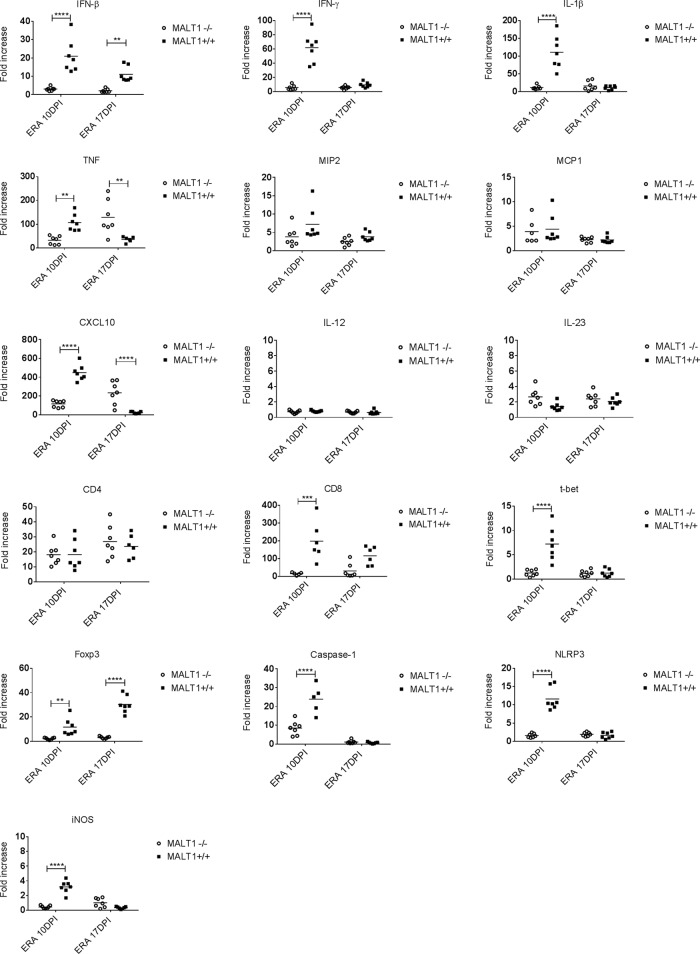
Defective expression of antiviral and inflammatory genes in the brains of ERA virus-infected MALT1^−/−^ mice. Quantitative RT-qPCR measurements of the indicated mRNA expression levels in brains of MALT1^+/+^ (*n* = 7) and MALT1^−/−^ littermate mice (*n* = 7) at 10 and 17 dpi are shown. The results are represented as the fold increases compared to noninfected MALT1^+/+^ and MALT1^−/−^ littermate mice, respectively. Differences in the fold increase at the same time point were determined by two-way ANOVA and Sidak's multiple-comparison test, and statistical differences between MALT1^+/+^ and MALT1^−/−^ mice are denoted by asterisks. Asterisks (****, ***, **, and *) represent *P* values of <0.0001, 0.001, 0.01, and 0.05, respectively.

To further analyze the increased virulence of ERA virus in MALT1-deficient mice, the virus-induced expression of several immune response genes in the brain was assessed. The mRNA levels of interferons (beta interferon [IFN-β] and IFN-γ), proinflammatory cytokines (tumor necrosis factor [TNF], interleukin-1β [IL-1β], IL-12, and IL-23), chemokines (MIP2, MCP1, and CXCL10), Th1-associated genes (IFN-γ, t-bet), T-reg-associated genes (Foxp3), inflammasome components (NLRP3, caspase-1), T cell markers (CD4 and CD8), and iNOS enzyme were measured in the brains of MALT1^+/+^ or MALT1^−/−^ mice at different time points after infection and compared to mRNA levels in the corresponding uninfected controls. As shown in Fig. 3, no upregulation of IL-12 could be observed. At 10 dpi, the expression of all other investigated genes was higher after ERA virus infection in infected mice compared to noninfected mice. In contrast, at comparable time points, virus-induced expression of the same genes was much less pronounced in the brains of MALT1^−/−^ mice, with the exception of MCP1, MIP2, CD4, and IL-23, which were expressed to a similar extent. The absence of Foxp3 expression reflects the known lack of thymic regulatory T cells in MALT1^−/−^ mice (35), whereas the absence of CD8 and IFN-γ indicates a defective cytotoxic T cell response. The antiviral IFN-β response and proinflammatory gene expression were also almost completely absent. At 17 dpi, virus-induced gene expression was in most cases less pronounced compared to 10 dpi, and differences between MALT1^+/+^ and MALT1^−/−^ were more gene dependent: the expression of IFN-β and Foxp3 was absent, whereas the expression of TNF and CXCL10 was significantly higher in the brains of MALT1^−/−^ mice than in MALT1^+/+^ mice. The latter results are in line with the high viral loads detected at 17 dpi in the brains of MALT1^−/−^ versus MALT1^+/+^ mice (Fig. 2). Together, these results suggest that at the early stage of infection (10 dpi), antiviral and inflammatory immune responses are severely disrupted in MALT1^−/−^ mice.

### MALT1 deficiency is associated with decreased infiltration and activation of inflammatory and immune cells in the brain at the presymptomatic phase of infection.

**FIG 4:**
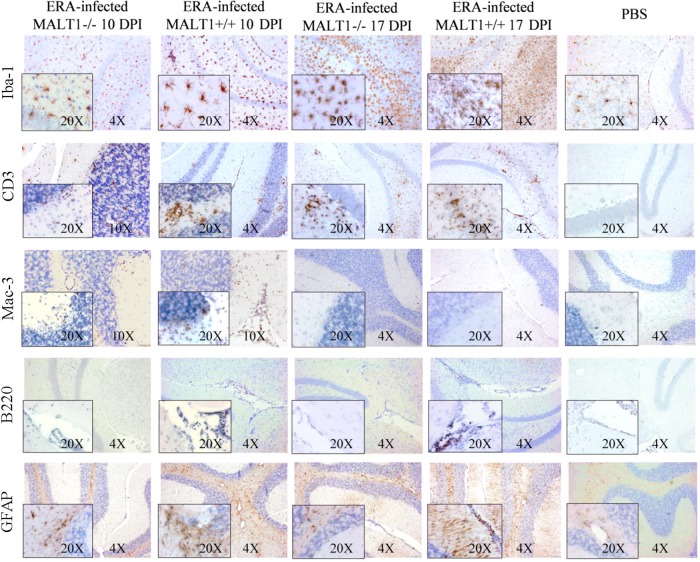
Reduced infiltration and activation of inflammatory cells in the brains of MALT1^−/−^ mice at 10 dpi with ERA virus. Immunohistochemical analysis of CNS sections from ERA virus-infected MALT1^+/+^ and MALT1^−/−^ mice at 10 and 17 dpi. PBS-inoculated MALT1^+/+^ mice were used as controls. Sections of the cerebellum and hippocampus are shown. Brain sections were immunostained for Iba-1 (microglial cells), CD3 (T cells), Mac-3 (macrophages), B220 (B cells), and GFAP (astrocytes). PBS-injected mice showed abundant inactive ramified microglial cells and astrocytes, but no B cell, macrophage, or T cell infiltration. At 10 dpi, ERA virus-infected MALT1^+/+^ mice showed activation of microglial and astroglial cells, infiltration of T lymphocytes and macrophages in the parenchyma, and infiltration of B cells around the blood vessels and choroid plexus. In MALT1^−/−^ mice, T lymphocyte and macrophage infiltration, as well as microglial activation, were reduced at 10 dpi. Moreover, B cells could not be observed around the blood vessels or the choroid plexus in MALT1^−/−^ mice. At 17 dpi, pronounced microglial activation and T lymphocyte infiltration were observed in both MALT1^+/+^ and MALT1^−/−^ mice. Macrophages were no longer visible. Astrogliosis increased further but was more pronounced in MALT1^+/+^ mice than in MALT1^−/−^ mice. Scale bars represent 20 μm (magnification, ×20), 50 μm (magnification, ×10), or 100 μm (magnification, ×4). Data are representative of two mice per condition.

To investigate whether the above-described defects in virus-induced cytokine and chemokine expression in MALT1^−/−^ mice were also associated with an altered recruitment and activation of inflammatory cells, immunohistological analysis for different inflammatory cell markers was performed on brain sections obtained at 10 and 17 dpi from ERA virus-infected MALT1^+/+^ and MALT1^−/−^ mice compared to phosphate-buffered saline (PBS)-inoculated control mice (Fig. 4). Microglial cells, which are the resident macrophages of the CNS, and astrocytes, which are the most abundant glial cell population, typically both respond to injury and infection by acquiring an activated phenotype, defined by morphological changes, migration, and proliferation (36, 37). Staining for the microglia cell marker Iba1 revealed an increased number of activated microglia at 10 and 17 dpi in MALT1^+/+^ mice compared to noninfected brains. Microglial activation is demonstrated by the typically bigger cell body and shorter and thicker branch processes. Resting microglial cells, observed mainly in noninfected mice, were characterized by their smaller cell bodies and long and ramified branch processes. In ERA virus-infected MALT1^−/−^ mice, the number of activated microglia cells was less pronounced at both 10 and 17 dpi compared to MALT1^+/+^ mouse brains. Astrocyte activation was also evident in ERA virus-infected MALT1^+/+^ mice, as demonstrated by enhanced glial fibrillary acidic protein (GFAP) immunoreactivity. At 10 dpi, GFAP staining was observed in the white matter of the cerebellum, whereas at 17 dpi, GFAP staining was observed in the white matter and in the outer layer of the gray matter. In ERA virus-infected MALT1^−/−^ mice, astrocyte activation in the outer layer of the gray matter was slightly reduced at 17 dpi.

Infiltration of CD3^+^ T cells and Mac-3^+^ macrophages was observed at 10 dpi in ERA virus-infected MALT1^+/+^ mice. The T cell infiltrates persisted at 17 dpi, whereas macrophage infiltrates were no longer visible. T cell and macrophage infiltration was much less pronounced in ERA virus-infected MALT1^−/−^ mice at 10 dpi, but similar levels of T cells were detected in ERA virus-infected MALT1^+/+^ and MALT1^−/−^ mice at 17 dpi. B220^+^ B cells were detectable around the blood vessels and in the choroid plexus, but not in the brain parenchyma, at 10 and 17 dpi in ERA virus-infected MALT1^+/+^ mice. In contrast, almost no B cells were detected in ERA virus-infected MALT1^−/−^ mice, either around blood vessels or in the choroid plexus. As expected, CD3^+^, B220^+^, or Mac-3^+^ staining was not detected in PBS-inoculated control mice. For all stainings, results were similar in cerebellum and hippocampus.

**FIG 5:**
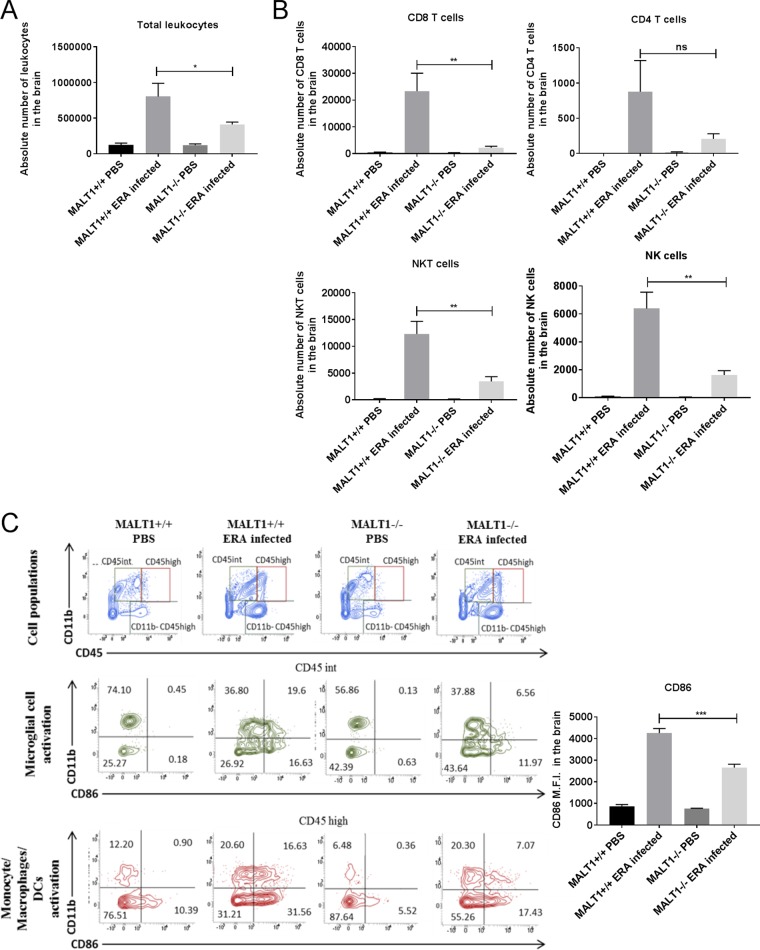
Flow cytometric analysis of immune cell activation and infiltration in the brains of ERA virus-infected mice. Immune cells were isolated from the brains of naive mice and infected mice (MALT1^+/+^ and MALT1^−/−^) at 10 dpi. (A) Absolute numbers of leukocytes present in the brain were first determined, and the total numbers of each cell type were determined by the percentage of marker expression on the total number of leukocytes. A significant decrease in total leukocytes was observed in ERA virus-infected MALT1^−/−^ mice compared to ERA virus-infected MALT1^+/+^ mice. (B) CD49b and CD3 markers were used to distinguish NK cells (CD49b^+^ CD3^−^), NKT cells (CD49b^+^ CD3^+^), and T cells (CD49b^−^ CD3^+^). CD8 and CD4 markers were also used. A significant decrease in NK cells, NKT cells, and CD8^+^ T cells was observed in ERA virus-infected MALT1^−/−^ mice compared to ERA virus-infected MALT1^+/+^ mice. (C) CD45 and CD11b markers were used to distinguish T cells (CD3^+^ CD11b^−^ CD45^high^), microglial cells (CD11b^+^ CD45^int^), and MMDCs (monocytes, macrophages, and DCs; CD11b^+^ CD45^high^). CD45^int^ cells were selected to analyze microglial activation by using CD11b and CD86, a costimulatory molecule expressed on activated antigen-presenting cells. CD45^high^ cells were selected to analyze monocyte, macrophage, and DC activation. Activation of microglial cells and MMDCs was determined by CD86 expression represented as the MFI. A significant decrease of CD86 MFI was observed in ERA virus-infected MALT1^−/−^ mice compared to ERA virus-infected MALT1^+/+^ mice, which corresponds to the dot plots. The dot plots are representative of five mice per condition. Statistical differences between MALT1^+/+^ and MALT1^−/−^ mice were determined using a Student *t* test . Asterisks (***, **, and *) represent *P* values of <0.001, 0.01 and 0.05, respectively.

**FIG 6:**
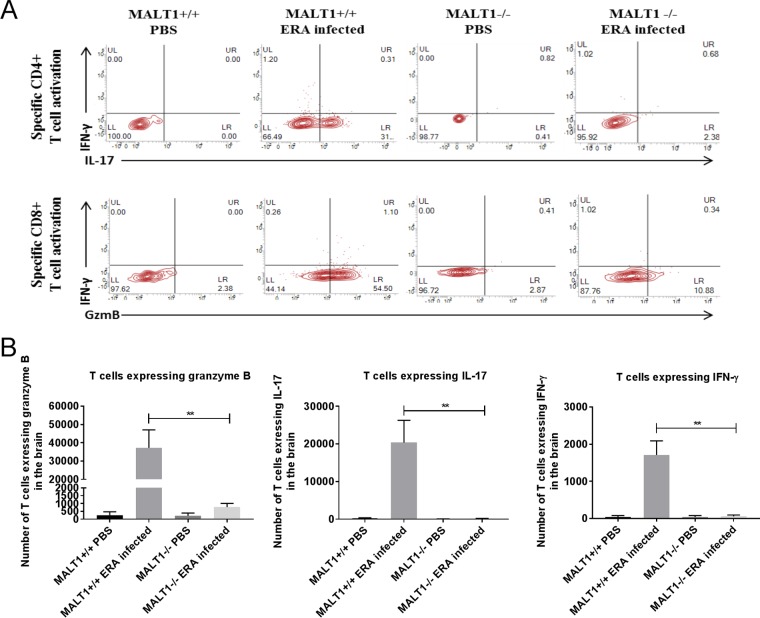
Reduced granzyme B, IL-17, and IFN-γ production in T cells from ERA virus-infected MALT1^−/−^ mice. Immune cells were isolated from the brains of naive mice and infected mice (MALT1^+/+^ and MALT1^−/−^) at 10 dpi and stained for the intracellular markers IFN-γ, IL-17, and granzyme B. T cells were gated for their phenotypic marker CD3. (A and B) FACS analysis revealed less production of IL-17, granzyme B, and IFN-γ in the T cells of ERA virus-infected MALT1^−/−^ mice compared to ERA virus-infected MALT1^+/+^ mice. The dot plots are representative of five mice per condition. (C) A significant decrease in T cells expressing IFN-γ, granzyme B, and IL-17 was observed in infected MALT1^−/−^ mice compared to infected MALT1^+/+^ mice. Statistical differences between MALT1^+/+^ and MALT1^−/−^ mice were determined by using a Student *t* test. Asterisks (***, **, and *) represent *P* values of <0.001, 0.01 and 0.05, respectively.

Inflammatory cell infiltration was also assessed by flow cytometric analysis of brain leukocytes at 10 dpi. As shown in Fig. 5A, strong leukocyte infiltration was observed in ERA virus-infected MALT1^+/+^ mice compared to noninfected mice. A comparatively lower number of leukocytes was detected in infected MALT1^−/−^ mice. Analysis of cell populations revealed strong infiltration of microglial cells (CD45^int^ CD11b^+^), monocytes, macrophages, and dendritic cells (MMDCs; CD45^high^ CD11b^+^), NK cells (CD49^+^ CD3^−^), NKT cells (CD49b^+^ CD3^+^), and T cells (CD49b^−^ CD3^+^), with a high proportion of CD8^+^ T cells and a few CD4^+^ T cells (Fig. 5B and C) in ERA virus-infected MALT1^+/+^ mice. Analysis of costimulatory markers such as CD86 showed that microglia and MMDCs were highly activated, which was confirmed by analyzing the mean fluorescence intensity (MFI) of the CD86 marker (Fig. 5C). In ERA virus-infected MALT1^−/−^ brains, the absolute number of total leukocytes was much lower compared to ERA virus-infected MALT1^+/+^ brains (Fig. 5A), which is also reflected by lower numbers of CD8^+^ T cells, NK cells, NKT cells, activated MMDCs, and activated microglia. No significant difference was observed for CD3^+^ CD4^+^ T cells (Fig. 5B and C), which is in line with equal CD4 mRNA expression in MALT1^+/+^ and MALT1^−/−^ mouse brains (Fig. 3). Furthermore, *in vitro* stimulation of leukocytes isolated from ERA virus-infected MALT1^+/+^ brains with inactivated ERA virus showed that T cells express IL-17, IFN-γ, and granzyme B, suggesting the recruitment of Th1, Th17, and CD8^+^ T cells. In contrast, leukocytes from ERA virus-infected MALT1^−/−^ mice produced significantly less IL-17, granzyme B, and IFN-γ (Fig. 6). Altogether, these data illustrate that MALT1 deficiency is associated with decreased infiltration and activation of inflammatory and immune cells in the brain upon ERA virus infection.

### MALT1 deficiency is associated with a defective antiviral humoral immune response.

**FIG 7:**
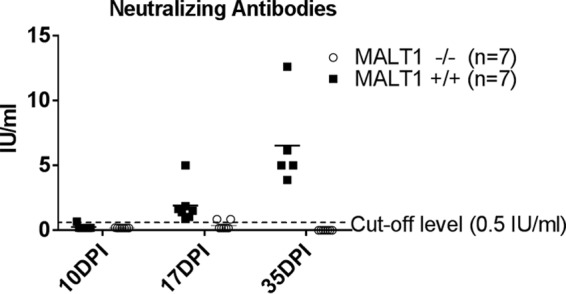
Defective humoral immune response in MALT1^−/−^ mice. In MALT1^+/+^ mice, antibody production was first detected in 1 out 7 mice at 10 dpi. At 17 dpi, neutralizing antibodies were detected in all MALT1^+/+^ mice (*n* = 7), and antibody levels increased further at 35 dpi (*n* = 7). Neutralizing antibodies were not detected in MALT1^−/−^ mice (*n* = 7) at 10 dpi and in 5 of 7 mice at 17 dpi. The two remaining MALT1^−/−^ mice had low levels of antibodies just above the cutoff at 17 dpi.

To investigate whether MALT1 deficiency also affects the humoral immune response against ERA virus we measured rabies virus neutralizing antibodies (VNA) in the serum. VNA were detected in the blood of MALT1^+/+^ infected mice at 17 dpi (1.90 ± 1.30 IU/ml). This time point was chosen because it is the endpoint of disease for MALT1^−/−^ mice. Titers increased further at 35 dpi (6.53 ± 3.12 IU/ml) (Fig. 7). However, most MALT1^−/−^ mice failed to produce VNA (<0.5 IU/ml). Low levels of VNA (0.85 IU/ml) could be detected in two MALT1^−/−^ mice at 17 dpi, but this had no impact on infection and disease outcome.

**FIG 8:**
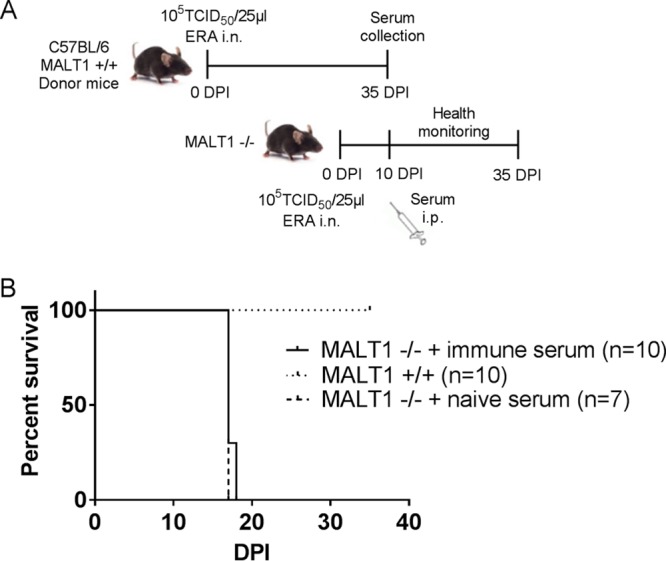
Transfer of neutralizing antibodies does not rescue MALT1^−/−^ mice. (A) Schematic overview of the experimental setup. MALT1^+/+^ mice were inoculated intranasally with the ERA virus and sacrificed at 35 dpi. Immune sera were collected, pooled, heat inactivated, and titrated prior to i.p. transfer to MALT1^−/−^ mice at 10 dpi (500 μl, 4.8 IU/ml). (B) Survival of infected mice. All MALT1^−/−^ mice developed severe disease and had to be euthanized, despite transfer of immune serum.

To further assess the role of antibodies in the protection against ERA virus in the brain, we tested whether the transfer of serum from immunized MALT1^+/+^ mice could infer protection in MALT1^−/−^ mice. Pooled immune serum (4.8 IU/ml, 0.5 ml/mouse intraperitoneally [i.p.]) was transferred to ERA virus-infected MALT1^−/−^ mice at 10 dpi, which corresponds to the time point when MALT1^+/+^ mice normally start to produce VNA after infection with ERA virus (Fig. 8A). Transfer of immune serum was unable to rescue the MALT1^−/−^ mice from lethal infection (Fig. 8B). These results suggest that antibodies alone are not sufficient to confer protection and that cell-mediated immunity is also necessary.

### MALT1 deficiency in T cells is sufficient to render ERA virus neurovirulent.

**FIG 9:**
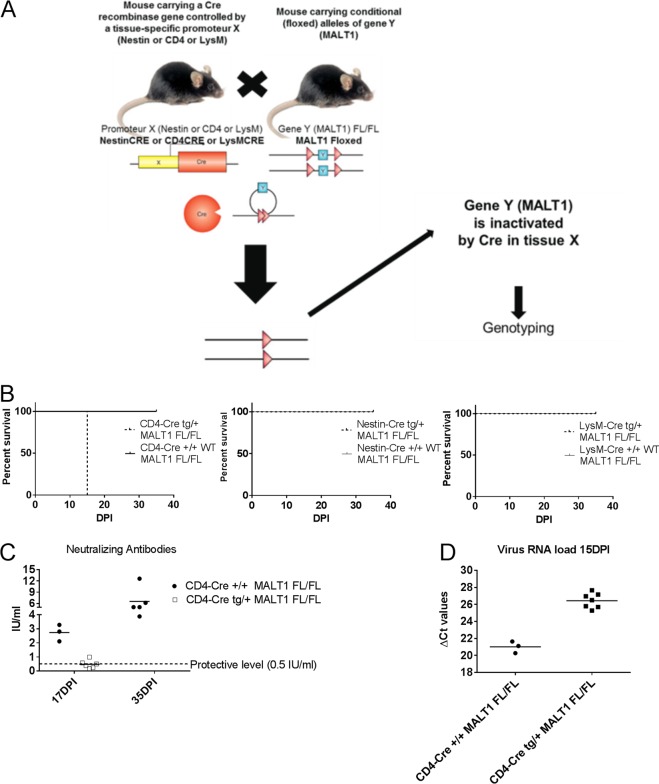
Impact of specific MALT1 deficiency in T cells, myeloid cells, or cells from neuroectodermal origin on ERA virus infection. (A) Schematic overview of conditional MALT1^−/−^ mouse generation. Mice expressing the CRE recombinase gene under the influence of the CD4, LysM, or Nestin promoter were crossed with MALT1^FL/FL^ mice to generate conditional mice lacking MALT1 in T cells, myeloid cells, or cells from a neuroectodermal origin, respectively. Conditional mice and their wild-type littermates were genotyped and selected for each experiment. (B) Survival of infected mice. Mice were infected intranasally with ERA virus and monitored for disease development and survival. Mice lacking MALT1 in cells from neuroectodermal origin (Nestin-Cre^tg/+^ MALT1^FL/FL^) and myeloid cells (LysM-Cre^tg/+^ MALT1^FL/FL^) developed only mild disease, comparable to their wild-type littermates. Mice lacking MALT1 in T cells (CD4-Cre^tg/+^ MALT1^FL/FL^) presented the same phenotype as the full MALT1^−/−^ and developed severe disease requiring euthanasia at 15 dpi. (C) Virus neutralizing antibodies in serum. Except for one, all CD4-Cre^tg/+^ MALT1^FL/FL^ mice failed to mount protective levels of neutralizing antibodies. (D) Viral RNA in total brain. All T cell-specific MALT1^−/−^ mice (CD4-Cre^tg/+^ MALT1^FL/FL^) presented a high viral load at 15 dpi, comparable to full MALT1^−/−^ mice.

MALT1^−/−^ mice were previously shown to have a normal number of T cells, but their capacity to be activated is impaired (15). We therefore hypothesized that MALT1 in T cells might be important to control ERA virus. This was investigated by using T cell-specific MALT1^−/−^ mice that were generated by crossing CD4-Cre mice with mice in which the MALT1 gene is flanked by two loxP Cre recombinase recognition sites (MALT1^FL/FL^) (Fig. 9A). In contrast to wild-type littermates (CD4-Cre^+/+^ MALT1^FL/FL^), infection of T cell-specific MALT1^−/−^ mice (CD4-Cre^tg/+^ MALT1^FL/FL^) with ERA virus led to severe disease and death between 15 and 17 dpi (Fig. 9B), similarly to the phenotype observed in MALT1 full-knockout mice (Fig. 1). In addition, we observed that most of the T cell-specific MALT1^−/−^ mice did not mount an antibody response (Fig. 9C). Indeed, only one mouse developed a low level of VNA (0.99 IU/ml). Accordingly, we also observed a significant increase of viral load in CD4-Cre^tg/+^ MALT1^FL/FL^ compared to their wild-type littermates (CD4-Cre^+/+^ MALT1^FL/FL^) (Fig. 9D). These results confirm the importance of the activation of CD4^+^ and CD8^+^ T cells through MALT1 to control ERA virus in the CNS. Theoretically, impaired signaling in neurons or myeloid cells could also be responsible for the increased pathogenicity of ERA virus in MALT1^−/−^ mice. Mice specifically lacking MALT1 in myeloid cells (LysMCre^tg/+^ MALT1^FL/FL^) or cells of neuroectodermal origin (neurons, astrocytes, or oligodendrocytes; Nestin-Cre^tg/+^ MALT1^FL/FL^) were therefore also generated and analyzed in similar infection experiments (Fig. 9A). Infection of Nestin-Cre^tg/+^ MALT1^FL/FL^ and LysMCre^tg/+^ MALT1^FL/FL^ mice yielded survival results comparable to those of wild-type littermates, excluding a role for MALT1 in neurons, astrocytes, oligodendrocytes, or myeloid cells (Fig. 9B). Together, our data demonstrate the importance of MALT1-mediated T cell activation to control infection with a live attenuated rabies virus in the brain.

### Pharmacological inhibition of MALT1 increases virulence of ERA virus.

**FIG 10:**
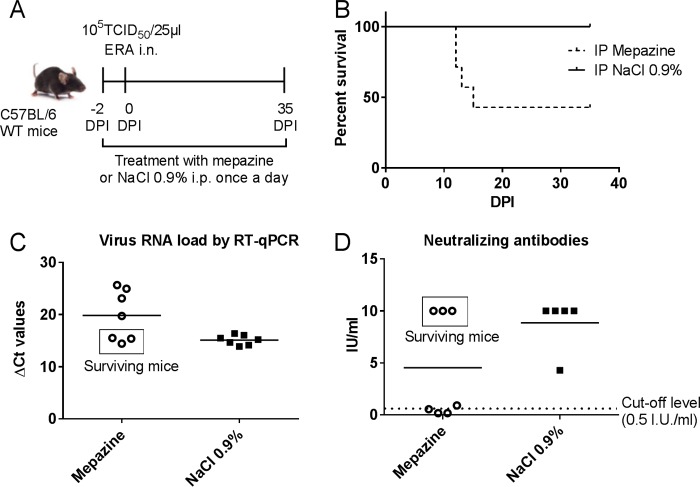
Treatment with mepazine in ERA virus-infected MALT1^+/+^ mice: impact on survival, viral load, and neutralizing antibody production. (A) Schematic overview of the experimental setup. MALT1^+/+^ mice were treated daily with mepazine (*n* = 7) or a control solution (0.9% NaCl) (*n* = 7) starting at day −2 before virus inoculation until the end of the experiment. Two days after the first treatment, mice were inoculated intranasally with ERA virus and monitored daily for signs of disease. (B) Survival curves. Four of seven mepazine-treated mice developed severe disease and had to be euthanized. Control mice and the remaining three mepazine-treated mice survived the infection. (C) Profile of viral RNA in total brains of mepazine-treated MALT1^+/+^ mice (*n* = 7) and control mice (*n* = 7) at sacrifice determined by RT-qPCR. Mepazine-treated mice with severe disease had higher viral loads. (D) Humoral immune response in mepazine-treated mice and control mice. Mepazine-treated mice with severe disease had no neutralizing antibodies. Control mice and the three surviving mepazine-treated mice had developed protective levels of neutralizing antibodies. The results were obtained from one experiment.

MALT1^−/−^ mice lack both MALT1 scaffold and MALT1 catalytic activities. To better delineate the specific role of MALT1 proteolytic activity in the control of ERA virus, we also tested the effect of inhibition of MALT1 catalytic activity by using the small compound inhibitor mepazine. Wild-type mice were treated daily with mepazine starting 2 days before virus inoculation and lasting until the late stage of disease (Fig. 10A). Control mice were treated with 0.9% NaCl. Some of the mepazine-treated mice (4/7) developed severe disease and had to be euthanized between 12 and 15 dpi, while the remaining mice developed only mild disease and survived the infection (Fig. 10B). At sacrifice, CNS viral loads of the mepazine-treated mice with severe disease were significantly higher than in untreated mice and reached levels similar to those observed previously in MALT1^−/−^ mice (Fig. 10C and 2B). At 35 dpi, surviving mepazine-treated mice and control mice presented similarly low viral loads (Fig. 10C). High levels of virus neutralizing antibodies were detected in the blood of the surviving mepazine-treated mice (>10 IU/ml) and control mice (8.86 ± 2.28 IU/ml), but not in the mepazine-treated mice that developed severe disease and died (0.45 ± 0.30 IU/ml) (Fig. 10D). These data demonstrate that MALT1 proteolytic activity is essential for a proper immune response and the control of rabies virus infection in the CNS.

## DISCUSSION

Although attenuated vaccines are often used to immunize humans and animals, there always remains a risk that these vaccines become pathogenic again either by acquisition of mutations or because of increased susceptibility of the host, as has been reported for attenuated rabies vaccines in wildlife (38–42). A better understanding of the immunological mechanisms that control attenuated rabies virus in the CNS is therefore of immediate interest. Our data demonstrate an important protective role for paracaspase MALT1-mediated signaling in the response to infection with the attenuated rabies virus ERA by mediating neuroinflammation at the early phase of infection. More specifically, we show that MALT1 deficiency in mice decreases cerebral immune responses at the early phase of infection, as evidenced by reduced microglial, astroglial, and T cell activation, less infiltration of macrophages and CD8^+^ T cells, and diminished expression of NF-κB regulated proinflammatory mediators in the brain at 10 dpi. At a later phase of infection (17 dpi), however, immune cell infiltration and activation were comparable between MALT1^+/+^ and MALT1^−/−^ mice, indicating that immune cells can still be activated and infiltrate the brain in ERA virus-infected MALT1^−/−^ mice, but with delayed kinetics compared to MALT1^+/+^ mice. In agreement with the curtailed immune response, MALT1^−/−^ mice developed severe disease, characterized by severe depression and paralysis and requiring human euthanasia, whereas MALT1^+/+^ mice developed only mild disease resolving at 11 dpi, characterized by transiently reduced activity. Similar results were observed in conditional knockout mice lacking MALT1 specifically in T cells. In contrast, mice lacking MALT1 specifically in neuroectodermal cells or myeloid cells survived the infection. Finally, pharmacological inhibition of MALT1 also increased the pathogenicity of the ERA virus. Together, our results demonstrate a key role for MALT1-dependent T cell activation in the control of attenuated rabies virus.

It is important to mention that T cell-specific MALT1^−/−^ mice lack MALT1 in both CD4^+^ and CD8^+^ T cells, since CD4-Cre is expressed during the double positive phase of T cell development. CD8^+^ T cells were the most recruited T cell population in the brain during ERA virus infection in wild-type mice. It has been shown before that CD8^+^ T cells are required for the early production of cytokines at the site of infection, contribute to the clearance of rabies virus from the CNS by enhancing IFN-γ production and the CNS inflammatory response, and kill infected cells by the release of granzyme B (43). Consequently, viral loads were found to be higher in CD8^+^ T cell-deficient mice (43). In MALT1^−/−^ mice, significantly fewer CD8^+^ T cells were recruited, whereas the number of CD4^+^ T cells remained the same as in MALT1^+/+^ mice. In contrast to others who have shown that rabies virus infection in CD8^+^ T cell-deficient mice remains sublethal (43), we observed lethal disease in MALT1^−/−^ mice, suggesting that decreased activation of CD4^+^ T cell subsets also contributes to the observed sensitization in MALT1^−/−^ mice. In this context, ERA rabies virus infection increased expression of the transcription factor t-bet and IFN-γ in the CNS, which is indicative for infiltration of CD4^+^ Th1 cells. Th1 cells were previously shown to be essential for the rapid clearance of attenuated rabies virus from the CNS (44), and the blunted expression of t-bet and IFN-γ in MALT1^−/−^ mice may thus be associated with the increased lethality. We also found evidence for increased Th17 infiltration and IL-17 production in the CNS of ERA rabies virus-infected mice. IL-17 in the brains of mice infected with a laboratory-attenuated rabies virus has previously been reported to enhance blood-brain barrier permeability and viral clearance (45–47). Interestingly, IL-17 levels in the brains of ERA virus-infected MALT1^−/−^ mice were lower than in MALT1^+/+^ mice, suggesting that MALT1 deficiency may decrease blood-brain barrier permeability, which on its turn may contribute to the reduced infiltration of inflammatory cells in the brain of infected MALT1^−/−^ mice. Loss of MALT1 has formerly been shown to also interfere with T cell infiltration and expression of IL-17 in experimental autoimmune encephalitis (EAE) in mice, a well-known model of multiple sclerosis (16, 17). In this case, MALT1 inhibition was shown to protect against EAE by blunting T-cell mediated autoimmunity.

Effective clearance of rabies virus requires the rapid production of neutralizing antibodies (43). ERA virus-infected MALT1^−/−^ mice failed to mount neutralizing antibodies. The same was true for conditional knockout mice lacking MALT1 specifically in T cells, illustrating that production of neutralizing antibodies against ERA virus is T cell dependent. Although rabies virus neutralizing antibodies are important for virus clearance, passive immunization of MALT1^−/−^ mice failed to rescue the mice. *In situ* production of antibodies by B cells in the brain (48) or active permeabilization of the blood-brain barrier is most likely required for antibodies to be effective against rabies virus (49). On the other hand, cellular immunity as described above is probably also required to control the virus in the brain. Together, these findings suggest that in the future, besides neutralizing antibodies, markers for cellular immunity should also be considered to assess vaccine efficacy and anti-rabies immunity.

**FIG 11:**
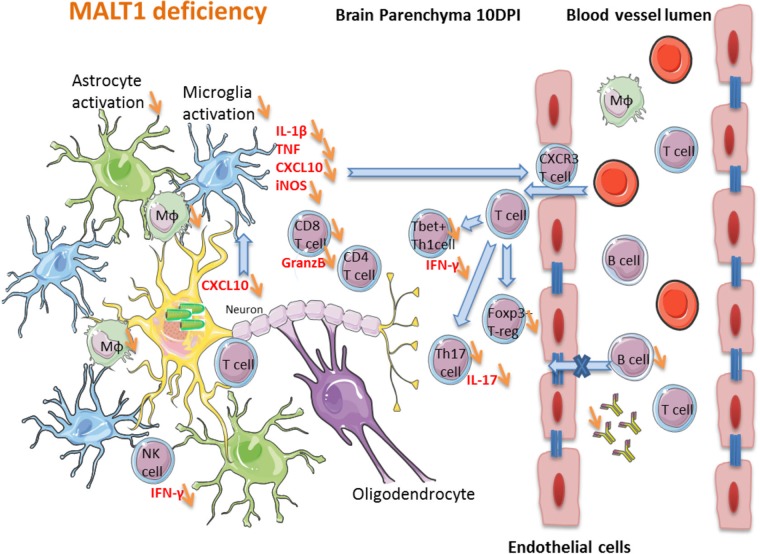
Hypothetical model of the impact of MALT1 inactivation on ERA virus-induced immune responses in the brain. In MALT1^+/+^ mice, virus-infected neuronal cells produce CXCL10 (as shown in other studies), leading to the activation of microglia and recruitment of CXCR3-expressing T cells and NK cells to the brain parenchyma. Activated microglia and astrocytes start to produce proinflammatory cytokines, iNOS and CXCL10, which amplifies the recruitment and activation of several immune cell types. Activated macrophages migrate to the cervical lymph nodes, where they serve as antigen-presenting cells for naive T cells and trigger the differentiation to effector T cells, mainly CD4^+^ Th1 and Th17 subsets and cytotoxic CD8^+^ T cells. Only activated T cells can infiltrate the brain. IFN-γ-producing CD8^+^ T cells are the main T cells recruited to the site of infection. Th1 cells and CD8^+^ T cells can further activate macrophages by IFN-γ production, and antigen-presenting cells can reactivate the T cells that have entered the brain. CD8^+^ T cells also produce granzyme B, mediating their cytotoxic activity and virus clearance. Th1 cells provide help for B cell activation and immunoglobulin production outside the brain, whereas Th17 cells produce IL-17, contributing to the activation of microglia and astrocytes, as well as enhancing blood-brain barrier permeability. The impact of MALT1 deficiency is represented by orange arrows. MALT1 deficiency in T cells reduces their activation and differentiation by antigen-presenting cells in the periphery, resulting in less effector T cells entering the brain. This not only results in less IFN-γ and IL-17 production but also lowers T cell help to activate B cells and CD8^+^ cytotoxic T cells, leading to defective production of virus neutralizing antibodies and reduced killing of virus-infected cells. As a consequence, viral load in the brain increases, exacerbating disease development.

A hypothetical model for the impact of MALT1 inactivation on ERA rabies virus-induced immune responses in the brain is depicted in Fig. 11. The high level of cytokines and inflammation in wild-type mice at 10 dpi with ERA virus, which coincides with some mild disease signs, together with the infiltration of activated T cells in the brain, might be crucial for effective control of the infection. MALT1^−/−^ mice presented less infiltration of activated T cells and inflammatory cytokine production in the brain and a significant reduction of CD8^+^ T cell, NK cell, and NKT cell infiltration. We propose that the reduction of immune cell infiltration in the brain, together with the lack of a peripheral humoral immune response, are at the origin of the higher viral loads and severe disease in MALT1^−/−^ mice.

The fact that MALT1 deficiency reduces neuroinflammation in the brain might also be of interest in the context of therapeutic modulation of other neurological pathologies involving neuroinflammation such as Alzheimer's disease (50), Parkinson's disease (51), multiple sclerosis (52), stroke (53), and neuropsychiatric diseases such as depression, schizophrenia, and autism (54, 55). In this context, the allosteric MALT1 inhibitor mepazine has already been shown to have a therapeutic effect in EAE (12). At the same time, however, our results indicate that MALT1 inhibitors can also increase the risk for severe virus infection in the brain or severe adverse effects upon vaccination with attenuated rabies viruses. Indeed, mepazine treatment of ERA virus-infected mice led to susceptibility to virulence of ERA virus in more than half of the mice. It should be mentioned that, similar to Nagel et al. (11), in our study mice were treated with 16 mg mepazine/kg (body weight) every 24 h. Considering the relatively short half-life of mepazine, a more frequent treatment schedule may still lead to stronger sensitizing effects for ERA infection. In addition to pharmacological MALT1 inhibition, mutations in MALT1 leading to MALT1 deficiency also may sensitize an attenuated rabies virus to neurovirulence. To date, a limited number of MALT1 deficient patients have been described (18–20), each presenting with severe combined immunodeficiency and being highly susceptible to fungal, bacterial, and viral infections. Moreover, other mutations may be less disruptive and only become apparent under certain conditions. It should be mentioned, however, that rabies vaccination in humans uses only inactivated vaccines. Therefore, the use of MALT1 inhibitors or MALT1 deficiency in combination with rabies vaccination in humans would not impose any risks. Since it remains to be seen whether our findings with an attenuated rabies virus also apply to other attenuated viruses, it is currently premature to generalize our findings to other attenuated virus vaccines. In future studies it will be interesting to also analyze the effect of MALT1 deficiency on infection with virulent rabies virus or other viruses.

## MATERIALS AND METHODS

### Mice.

MALT1^−/−^ mice were generated and generously provided by the team of Tak Mak (15). Mice were backcrossed for more than 10 generations into the C57BL/6 background and were intercrossed to generate MALT1^+/+^, MALT1^+/−^, and MALT1^−/−^ offspring. Mice lacking MALT1 specifically in T cells were generated by crossing the CD4-Cre line (56) and mice in which the *MALT1* gene is flanked by two loxP Cre recombinase recognition sites (MALT1^FL/FL^) on a C57BL/6 background. MALT1^FL/FL^ mice were originally derived from the EUCOMM Malt1^tm1a(EUCOMM)Hmgu^ strain. Mice lacking MALT1 specifically in cells from neuroectoderm origin (neurons, astrocytes, and oligodendrocytes) were generated by crossing the Nestin-Cre line (57) and MALT1^FL/FL^. Mice lacking MALT1 specifically in myeloid cells were generated by crossing the LysM-Cre line (58) and MALT1^FL/FL^. All mice were bred and housed in filter top cages in temperature-controlled, air-conditioned facilities with 14/10-h light/dark cycles and food and water *ad libitum* and used at the age of 6 to 12 weeks. All experimental procedures were approved by the Local Ethical Committee of the Scientific Institute of Public Health (WIV-ISP) and the Veterinary and Agrochemical Research Center (CODA-CERVA) (advice 070515-05).

### Genotyping.

MALT1^−/−^, MALT1^+/+^, and MALT1^+/−^ mice were genotyped using the primers P08_11 (GGG TAC ATC ATG GCC TGA ACA GTT G), P08_12 (TCC ACT CCA GCT CTT CTG CTA ACCAG), and P08_13 (GGG TGG GAT TAG ATA AAT GCC TGC TC), which resulted in PCR products of 900 bp for MALT1^+/+^, 1,000 bp for MALT1^−/−^, or both for heterozygous MALT1^+/−^ mice. The MALT1 Flox-allele was detected with the primers MALTcKO-F (GTT TCT CAG GTC TTT AGT TCA TGT C), CoMLT-3-R (TAT ACT CTA CAT CTC CAT GGT), or MALTcKO-R (TTG TTT TGC AGA TCT CTG CC), which resulted in PCR products of 280 bp for MALT1^+/+^, 400 bp for MALT1^FL/FL^, or 345 bp for MALT1^−/−^ mice. The Cre allele was detected with the primers Cre-F (TGC CAC GAC CAA GTG ACA GCA ATG) and Cre-R (AGA GAC GGA AAT CCA TCG CTC G), producing a 374-bp PCR fragment. Transgenic mice were identified by PCR analysis of genomic DNA extracted from tails, and amplification of the selected fragments was performed using the GoTaq G2 DNA polymerase (Promega, Madison, WI) master mix according to a typical PCR program: denaturation for 5 min at 95°C; followed by 35 to 40 cycles of 30 s at 95°C, 30 s at 55 to 60°C, and 60 s at 72°C; and then 10 min at 72°C for final elongation. Fragments were visualized on a 2% agarose gel.

### Virus.

The attenuated Evelyn-Rotnycki-Abelseth (ERA) virus, used as an oral vaccine for immunization of wild life, was obtained from the American Type Culture Collection (reference no. VR322) (38). Viral stocks were produced in baby hamster kidney-21 (BHK-21) cells (Deutsche Sammlung von Mikroorganismen und Zellkulturen, GmbH, Braunschweig, Germany). The lysates of infected cell cultures were centrifuged at 20,000 × *g* for 20 min at 4°C, and supernatants were stored at −80°C.

### Virus titration.

Infectious rabies virus particles were titrated by an endpoint dilution assay in BHK-21 cells. The measure of infectious virus titer was expressed as 50% tissue culture infective dose (TCID_50_)/ml and represents the amount of virus per ml that gives rise to infection in 50% of inoculated tissue culture cells. Virus titration was performed according to the Manual of Diagnostic Tests and Vaccines for Terrestrial Animals (Office International des Epizooties, 2008).

### Virus inoculation, clinical follow-up, euthanasia, and sampling.

Mice were inoculated intranasally with ERA virus using 10^5^ TCID_50_ in 25 μl of PBS, during brief anesthesia with isoflurane, as described by Rosseels et al. (59). Intranasal inoculation of virus was chosen since administration by this route results in a quick invasion of the CNS. Mice were monitored once a day for signs of disease throughout the experiment until 35 days postinoculation (dpi). A cumulative daily clinical score per mouse was obtained by adding the scores for each parameter and ranged from 0 (no disease) to 9 (severe brain disease). Disease signs were scored as follows: no signs = 0, rough hair coat = 1, depression/isolation from the group = 1, hunched back = 1, slow movement = 1, paresis in the front paws = 1, uncoordinated movements = 1, no spontaneous movements = 1, paralysis of hind legs = 1, and no response to external stimuli (end stage) = 1. Disease progression is represented by a curve of the mean cumulative score per group. Once mice reached a minimum score of 6, or at predetermined time points after virus inoculation (10 dpi, incubation phase; 17 dpi, end stage of disease), mice were euthanized with an overdose of ketamine (100 mg/kg; Ceva, Brussels, Belgium) and xylazine (9.9 mg/kg; Rompun 2; Bayer Healthcare, Kiel, Germany) (300 μl/mouse, i.p.). Upon terminal anesthesia, the blood was flushed from the circulatory system by transcardial perfusion with a PBS solution. Brains were collected and divided into two halves according to a longitudinal section. One half was stored at −80°C for further analysis by real-time RT-qPCR or FAT, and the other half was submerged in 4% formaldehyde for fixation and further immunohistochemical analysis. Serum was collected from mice at different time points postinfection and upon euthanasia for titration of virus-neutralizing antibodies. Mice that did not develop disease signs were terminally anesthetized, perfused, and euthanized at 35 dpi.

### RNA extraction and real-time RT-qPCR assay for quantification of viral RNA and inflammatory gene expression.

Seven mice per group were euthanized at each indicated time point. RNA extraction was performed using the RNeasy minikit (Qiagen, Hilden, Germany) according to the manufacturer's instructions. RNA concentration was calculated using Nano Vue spectrophotometry (GE Healthcare, Bucks, UK) and 100 ng was used for reverse transcription. Reverse transcription and qPCR were performed as described by Rosseels et al. (59). Two primers (forward and reverse) located in the nucleoprotein N genome region were used for quantification of viral RNA. All primer sequences are listed in Table 1. All samples were analyzed in duplicates. Amplification was performed on an iCycler iQ (Bio-Rad) in a 96-well optical plate format according to the following program: 2 min at 95°C, followed by 45 cycles of 20 s at 95°C and 30 s at 62°C. A melting-curve analysis was performed in order to verify the specificity of amplicons. Expressions of inflammatory genes were normalized using the cellular 18S rRNA housekeeping gene. Quantification of immune gene expression was performed using the comparative ΔΔ*C_T_* method. The fold change was calculated as 2^−ΔΔ*CT*^, with ΔΔ*C_T_* = Δ*C_T_*_ sample_ – Δ*C_T_*_ ctrl_. Δ*C_T_*_ sample_ = *C_T_*_ gene_ – *C_T_*_ 18S_ and Δ*C_T_*_ ctrl_ = *C_T_*_ ctrl_ – *C_T_*_ 18S_, with *C_T_*_ ctrl_ corresponding to the cycle threshold (*C_T_*) of gene expression in noninfected brains. For the detection of the viral N protein's gene, delta cycle threshold (Δ*C_T_*) values were calculated using the following formula: Δ*C_T_* = *C_T_*_ ref_ – *C_T_*_ sample_, with *C_T_*_ ref_ equal to 45, which is the maximum number of cycles of this qPCR program. A Δ*C_T_* value of 0 means there was no detection of viral RNA.

**TABLE 1:**
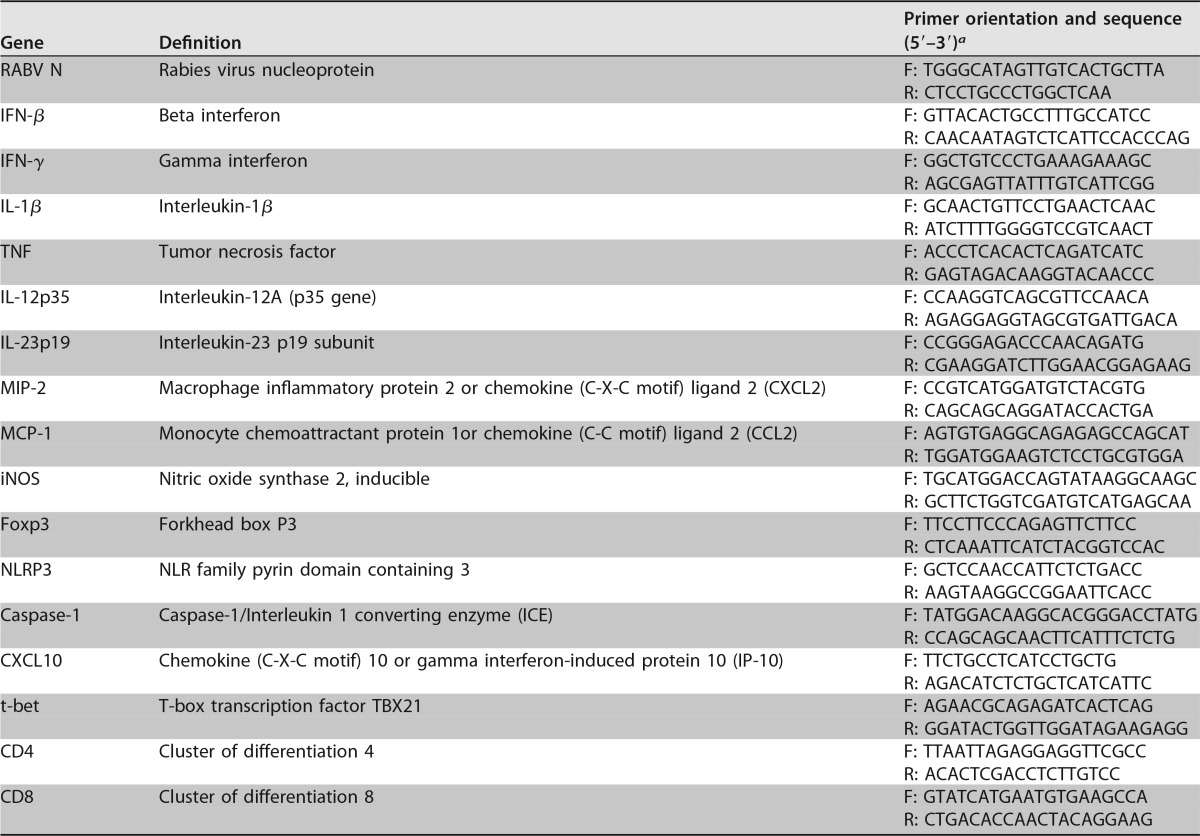
Primers used in real-time PCR

^*a*^ F, forward; R, reverse.

### Titration of neutralizing antibody by RFFIT.

Mouse blood samples were used for measurement of neutralizing antibodies by using the rapid fluorescent focus inhibition test (RFFIT) according to the *Manual of Diagnostic Tests and Vaccines for Terrestrial Animals* (Office International des Epizooties, 2008). Neutralizing antibody titers are expressed as international units (IU)/ml in reference to “The Second International Standard for Anti-Rabies Immunoglobulin,” purchased from the United Kingdom National Institute for Biological Standards and Control. Serum titers of 0.5 IU/ml or higher are an indication of seroconversion.

### Detection of nucleocapsid antigen of rabies virus by FAT.

The fluorescent antigen test (FAT) was performed according to the *Manual of Diagnostic Tests and Vaccines for Terrestrial Animals*. Brain smears were fixed with 75% acetone for 30 min at −20°C and incubated with fluorescein isothiocyanate-coupled anti-nucleocapsid rabbit antibody for 30 min at 37°C.

### Immunohistological analysis of mouse brains for expression of Iba-1, GFAP, B220, CD3, and Mac-3.

Virus-inoculated mice and PBS-inoculated mice were transcardially perfused with PBS. Brains were removed, immersed in 4% paraformaldehyde, dehydrated, and embedded in paraffin blocks. Sections of 2 μm were obtained, and microglial cells, astrocytes, B cells, T cells, and macrophages were detected using specific antibodies: rabbit anti-Iba-1 (Wako Chemicals, Fuggerstrasse, Germany), rabbit anti-GFAP (Dako/Agilent Technologies, Santa Clara, CA), rat anti-B220 (eBioscience/Thermo Fisher Scientific, Waltham, MA), rabbit anti-CD3 (Dako/Agilent Technologies), and rat anti-Mac-3 (BD Pharmingen, San Diego, CA), respectively. Sections were rehydrated and incubated in 10 mM citrate buffer for 10 min at 95°C. To remove endogenous peroxydase, tissue sections were treated with 3% H_2_O_2_ in methanol. To block nonspecific reactions, each section was treated with 5% goat serum in antibody diluent (Dako/Agilent Technologies) for 30 min. Primary antibody was diluted in 5% goat serum in antibody diluent with the dilution depending on the staining realized (Iba-1, 1/1,000; CD3, 1/200; MAC-3, 1/250; B220, 1/1,000; GFAP, 1/200,000) and incubated on brain sections overnight at 4°C. Goat anti-rabbit (Dako/Agilent Technologies) or goat anti-rat (BD Pharmingen) secondary antibodies conjugated to biotin were diluted at 1:500 in block buffer and added for 45 min-1 h to the sections. Drops of avidin/biotin-based peroxidase system Vectastain Elite ABC (Vector Laboratories, Burlingame, CA) were added to each section for 30 min. Finally, antibody was visualized using 3,3′-diaminobenzidine (DAB; Dako/Agilent Technologies) until specific staining appeared; slides were counterstained with hematoxylin, dehydrated, and mounted with a xylene-based mounting medium. Sections without primary antibody were taken as controls.

### Immunophenotyping of leukocytes.

Immune cells were extracted from the brain at 10 dpi as described previously (60). In brief, brains were collected, and single-cell suspensions were prepared by gently pressing the organs in Hanks balanced salt solution (HBSS) through a nylon mesh (mesh pore size, 100 μm). After centrifugation, the cells were placed in collagenase-DNase for 1 h. After washing, the suspension was centrifuged in 25% Percoll (Sigma-Aldrich, St. Louis, MO), the myelin layer was removed, and the cells were suspended in HBSS containing 10% fetal calf serum (FCS). Red blood cell lysis buffer was added to the cell pellet to eliminate erythrocytes. Cells were washed twice with fluorescence-activated cell sorting (FACS) buffer (PBS containing 10% FCS and 0.1% NaN_3_) and incubated with FcγII/III receptor blocking anti-CD16/CD32 (clone 2.4G2; BD Biosciences, San Jose, CA) for 15 min at 4°C. The cells were then incubated for 30 min at 4°C protected from light with viability dye (Thermo Fisher) and the appropriate dilutions of the following antibodies: Alexa Fluor 488-anti CD11b (clone M1/70), Brilliant Violet-421 anti-CD45 (clone 104), PE-anti-CD49b (clone DX5), APC-Cy7-anti-CD3 (clone 145-2C11), PerCP-Cy5.5-anti-CD8 (clone 53-6.7), APC-anti-CD4 (clone GK1.5), or PE-Cy7-anti-CD86 (clone GL1). The stained cells were washed twice in FACS buffer, fixed with 4% paraformaldehyde, and analyzed in a BD FACSVerse flow cytometer. Data were analyzed using BD FACSuite software. Antibodies were from BD Pharmingen or Thermo Fisher.

### Activation of T cells.

For analysis of T cell activation in the brain, collected leukocytes were cultured in the presence of 1 μg/ml anti-CD28 (clone 37.51), 1 μg/ml anti-CD49d (clone 9C10 [MFR4.B]), 10 μg/ml ERA inactivated antigens, and brefeldin A (Thermo Fisher) for 5 h at 37°C. The cells were then harvested, washed twice, and suspended in FACS buffer containing FcγII/III receptor blocking anti-CD16/CD32 (clone 2.4G2) for 15 min at 4°C. The cells were stained with viability dye and a cocktail of extracellular antibodies (Brilliant Violet-421 anti-CD45 [clone 104], APC-Cy7-anti-CD3 [clone 145-2C11], APC-anti-CD4 [clone GK1.5], and PerCP-Cy5.5-anti-CD8 [clone 53-6.7]). The cells were then washed twice with FACS buffer and permeabilized using Cytofix/Cytoperm kit (BD Pharmingen). Cells were subsequently suspended in 1× PermWash buffer (BD Pharmingen) containing a cocktail of PE-anti-IFN-γ (clone XMG1.2), Alexa Fluor 488-anti-IL-17A (clone TC11-18H10), and PE-Cy7-GranzymeB (NGZB). Isotype control and Fluorescence Minus One (FMO) control staining were performed in parallel. Cells were then washed twice and fixed with 4% paraformaldehyde. Samples were examined using a BD FACSVerse flow cytometer and analyzed with BD FACSuite software. The antibodies were obtained from BD Pharmingen or Thermo Fisher.

### Serum transfer by i.p. injection.

Sera were collected at 35 dpi from immune C57BL/6 mice infected with 10^5^ TCID_50_ of ERA virus/25 μl. MALT1^−/−^ mice received 500 μl of serum i.p. on day 10 after virus challenge. The sera of naive mice were used as a control. Mouse sera were heat inactivated prior to administration (30 min at 56°C).

### Treatment with mepazine.

Mice were randomly treated with either mepazine or 0.9% NaCl. Mepazine (ChemBridge) was solubilized in NaCl 0.9% at a concentration of 2 mg/ml. Mice were injected i.p. daily with 200 μl (16 mg/kg) starting 2 days before ERA virus inoculation until the end of the experiment. Mice were monitored once a day for signs of disease throughout the experiment.

### Statistical analyses.

Statistical analysis was performed using the Student *t* test for unpaired data or the two-way analysis of variance (ANOVA), followed by a Sidak multiple-comparison test in GraphPad Prism7. A log-rank test was used to analyze Kaplan-Meier survival curves.
